# TOWARD A NETWORK OF CHANGE: PROMOTING AGE-FRIENDLY COMMUNITIES FROM THE INSIDE OUT

**DOI:** 10.1093/geroni/igz038.095

**Published:** 2019-11-08

**Authors:** Shellae Versey, Emily Greenfield

**Affiliations:** 1 Wesleyan University, Middletown, Connecticut, United States; 2 Rutgers University, New Brunswick, New Jersey, United States

## Abstract

The experience of aging is often framed by the communities in which we live, work and play. At the same time, these communities are also impacted by individuals as they age in place. This symposium presents research using community-partnered methods to highlight the agency of local actors—including older residents themselves—as they work to change their local communities. At the broadest level of geographic scale, Black illustrates how information from population surveys with older adults can be leveraged to mobilize public-private partnerships from the local to state level to advance policy and practice on housing and transportation to support aging in place. Focusing regionally in New Jersey, Greenfield and Reyes analyze longitudinal, qualitative interview data from leaders of age-friendly community initiatives to develop empirically-grounded theory on the range of roles of older adults in aging-friendly community change processes. The final two papers present depth in understanding how older adults actively construct their own sense of community vis-à-vis more micro-social processes. Yeh uses Photovoice methodology to understand how older adults aging in place manage the societal trends of urbanization and social inequalities as they manifest within their own city. Versey examines how older adults aging in place in the context of neighborhood gentrification mobilize networks to preserve a community “sense of place” when sociocultural resources are displaced. Consistent with a community gerontology framework, the presentations demonstrate how community-level dynamics around aging are shaped not only by macro-social influences, but also micro-social interactions including, and sometimes initiated by, older residents themselves.

